# How the World Bank's Pandemic Fund can address health inequities

**DOI:** 10.1002/puh2.138

**Published:** 2023-11-10

**Authors:** Yusuff Adebayo Adebisi, Aminat Olaitan Adebayo, Nafisat Dasola Jimoh, Olusegun Ayo Ade‐adekunle, Mba Oluebube Mercy, Sarah Kuponiyi

**Affiliations:** ^1^ Nuffield Department of Population Health University of Oxford Oxford Oxfordshire UK; ^2^ Department of Agricultural Extension and Rural Development, Faculty of Agriculture University of Ibadan Ibadan Nigeria; ^3^ Federal University of Technology Minna Minna Niger Nigeria; ^4^ Faculty of Pharmacy Olabisi Onabanjo University Ikenne Ogun Nigeria; ^5^ David Umahi Federal University Teaching Hospital Uburu Ebonyi Nigeria; ^6^ Liverpool School of Tropical Medicine Liverpool UK

The Pandemic Fund is established to strengthen pandemic prevention and response capabilities in low‐ and middle‐income countries and address critical gaps through investments and technical support at the national, regional and global levels. It is governed by a Board comprising representatives from sovereign contributors, non‐sovereign contributors, sovereign co‐investors and civil society organizations. This Fund represents a multi‐stakeholder global partnership, with its Secretariat hosted by the World Bank, which also acts as a trustee for the fund, and with technical leadership from the World Health Organization [1]. The recent announcement on 3 March 2023 of a $300 million allocation for the Pandemic Fund's initial round of funding represents a stride towards enhancing prevention, preparedness and response initiatives for public health emergencies in low‐and middle‐income countries [2]. It is, however, pertinent that the Pandemic Fund bolsters inclusive and holistic strategies that prioritize the needs of vulnerable communities in order to effectively tackle health disparities and foster equity.

The priority areas specified in the Call for Proposals – namely disease surveillance, laboratory systems and workforce capacity – are indisputably crucial components of preparedness and response efforts [2]. Although these elements play a foundational role in strengthening health infrastructures and promoting early outbreak detection, there is a pressing need for a more comprehensive discussion on health system strengthening. This should emphasize not only the infrastructure but also the access to quality care and the equitable distribution of resources. These components not only support early detection but also underpin a dependable emergency health response. It is imperative, however, to recognize that marginalized groups – such as ethnic minorities, the economically disadvantaged, people with disabilities and residents of remote areas – often suffer disproportionately during health crises. In the wake of health emergencies like COVID‐19, the pronounced health disparities and the severe implications for these vulnerable populations became glaringly evident [3, 4]. These groups encountered a myriad of challenges, from higher infection and mortality rates to limited healthcare access, all of which magnified existing socio‐economic inequalities [5].

The necessity of fully engaging minority communities in pandemic preparedness and response initiatives cannot be overstated. Such engagement is paramount for crafting strategies that address the unique vulnerabilities and barriers these communities face in health emergencies and has been widely recognized in the literature for HIV/AIDS [6]. The Pandemic Fund has the opportunity to facilitate this vital inclusion by prioritizing and funding initiatives that highlight active engagement with these communities. One approach for this engagement could involve robust collaboration with local organizations, grassroots movements and community leaders that have a deep understanding of the specific needs, barriers and socio‐cultural dynamics within these communities. This could ensure that funded initiatives are appropriately tailored to address distinct health and socio‐economic challenges faced by these marginalized groups. Embracing an intersectional approach would underscore the diverse challenges these communities face, providing a blueprint for more targeted interventions. Moreover, involving community stakeholders in the design and implementation of interventions ensures a degree of cultural sensitivity and appropriacy that might be lacking in top‐down initiatives. The active integration of the viewpoints of marginalized groups in decision‐making processes is also crucial. This can be achieved by involving representatives from these communities in consultation processes, planning meetings and review committees. Hence, the Pandemic Fund can ensure that its initiatives align with the priorities, values and needs of the communities they are meant to serve.

Investing in community‐based initiatives is another critical strategy for promoting local ownership of pandemic preparedness and response efforts. For instance, supporting community health workers can be particularly effective, given their unique position within the communities they serve. These workers understand the local needs, challenges and cultural contexts, which equips them to deliver services and information in a way that resonates with the community. A practical example is illustrated by the role of community health workers in the fight against the Ebola virus in West Africa. As documented in the literature [7], their local expertise and community trust played a crucial role in breaking the chain of transmission by rapidly identifying and reporting suspected cases, as well as educating community members about preventive measures. Their presence not only improved health outcomes but also bridged the gap between formal health systems and remote or marginalized communities, ensuring a more equitable health response.

Furthermore, experts suggest that the Pandemic Fund can enhance its effectiveness by learning from and leveraging the proven strategies and proficiencies of established global health organizations and initiatives [8]. For example, the Global Fund's success in addressing HIV/AIDS, Malaria and Tuberculosis in developing countries offers valuable insights. The Pandemic Fund could adopt similar strategies for reaching key populations and ensuring that they are not left behind. These established entities have laid out pathways and best practices, having achieved notable successes in health challenges across various regions. Leveraging their expertise, strategies and networks would not only allow for enhanced impact but also provide opportunities for synergy, reducing duplication of efforts and ensuring a unified approach towards addressing health inequities and promoting health equity. Alongside this, there is a need for the Pandemic Fund to bolster efforts aimed at enhancing data collection and analysis systems. Accurate, robust data is vital for illuminating the unique needs and challenges faced by marginalized populations. The fund can help develop and implement mechanisms for gathering and analysing such data, ensuring that these populations are adequately represented in all stages of pandemic preparedness and response. This data could, in turn, be used to inform targeted interventions and policies that specifically address these needs.

In addressing critical observations related to the World Bank's Pandemic Fund, it is paramount to engage with prevailing concerns that have been brought to the fore by various stakeholders and scholars. Central to these apprehensions is the Fund's governance structure, operational mechanisms and the breadth of its mandate [8]. A salient critique lies in the alleged exclusion of significant regional stakeholders, such as the Africa CDC as an implementing entity [9], which raises questions about representation and inclusivity in the decision‐making processes of the Fund. Such exclusions might inadvertently limit the scope and efficacy of the Fund's interventions, especially in regions where localized insights are crucial for effective response strategies. Additionally, there is a discernible gap in the fund's provision for response activities, a limitation that could hamstring immediate and comprehensive interventions in the face of emerging health crises [8]. Furthermore, the call for the adoption of a global public investment model amplifies the need for diversified funding mechanisms [10]. Such an approach would not only augment the financial pool available for pandemic interventions but also promote a sense of shared responsibility and commitment across nations. Governance, in this context, is not merely an administrative function but serves as the bedrock ensuring that the Fund's objectives are met holistically and equitably. Emphasizing sustainability in the Pandemic Fund's strategies is also essential. Fostering resilience, strengthening health infrastructures and endorsing self‐sufficiency within LMICs provide a long‐term vision for combating future health crises [4]. Equally crucial is the implementation of a rigorous evaluation framework for the initiatives funded by the Pandemic Fund. Regularly measuring the impact of these initiatives on health equity is essential for ensuring accountability, learning from successes and failures and informing the direction of future investments. By adopting a framework for evaluating its activities, the Pandemic Fund can continuously refine its strategies and approaches to maximize its effectiveness and impact.

In an interconnected world, pandemics do not respect national borders, and the health of one community can have far‐reaching implications for the health of others. Ensuring that all populations, particularly marginalized communities, have the resources and support needed to prevent and respond to pandemics is crucial for global health security and the overall well‐being of our societies.

## AUTHOR CONTRIBUTIONS

This article was conceptualized by Yusuff Adebayo Adebisi. Yusuff Adebayo Adebisi and Nafisat Dasola Jimoh wrote the first draft, whereas Aminat Olaitan Adebayo, Olusegun Ayo Ade‐adekunle, Mba Oluebube Mercy and Sarah Kuponiyi revised the first draft critically for important intellectual content.

## CONFLICT OF INTEREST STATEMENT

Yusuff Adebayo Adebisi is an Editorial Board member of Public Health Challenges and co‐author of this article. To minimize bias, he was excluded from all editorial decision‐making related to the acceptance of this article for publication.

## FUNDING INFORMATION

This article did not receive any specific grant from funding agencies in the public, commercial or not‐for‐profit sectors.

## ETHICS STATEMENT

None.

## Data Availability

Not applicable.

## References

[1] Boyce MR , Sorrell EM , Standley CJ . An early analysis of the World Bank's Pandemic Fund: a new fund for pandemic prevention, preparedness, and response. BMJ Glob Health. 2023;8(1):e011172. doi:10.1136/bmjgh-2022-011172 PMC9815014 36599499

[2] World Bank . The Pandemic Fund . World Bank. Accessed on June 1, 2023. https://www.worldbank.org/en/programs/financial‐intermediary‐fund‐for‐pandemic‐prevention‐preparedness‐and‐response‐ppr‐fif/funding‐opportunities

[3] Adebisi YA , Ekpenyong A , Ntacyabukura B , et al. COVID‐19 highlights the need for inclusive responses to public health emergencies in Africa. Am J Trop Med Hyg. 2020;104(2):449–452. doi:10.4269/ajtmh.20-1485 33331263 PMC7866309

[4] Evaborhene NA , Udokanma EE , Adebisi YA , et al. The pandemic treaty, the pandemic fund, and the global commons: our scepticism. BMJ Glob Health. 2023;8(2):e011431. doi:10.1136/bmjgh-2022-011431 PMC9980354 36854490

[5] Barron GC , Laryea‐Adjei G , Vike‐Freiberga V , et al. Lancet commission on COVID‐19: task force on humanitarian relief, social protection and vulnerable groups. Safeguarding people living in vulnerable conditions in the COVID‐19 era through universal health coverage and social protection. Lancet Public Health. 2022;7(1):e86–e92. doi:10.1016/S2468-2667(21)00235-8 34906331 PMC8665842

[6] Mehrotra A , Davis DA , Evens E , White B , Wilcher R . The importance of key population community engagement and empowerment in HIV programming: insights from a global survey with local implementing partners. J Glob Health Reports. 2020;4:e2020044.

[7] McMahon SA , Ho LS , Brown H , Miller L , Ansumana R , Kennedy CE . Healthcare providers on the frontlines: a qualitative investigation of the social and emotional impact of delivering health services during Sierra Leone's Ebola epidemic. Health Policy Plan. 2016;31(9):1232–1239. doi:10.1093/heapol/czw055. Epub 2016 Jun 8. 27277598 PMC5035780

[8] Saavedra J , Faviero GF , Kurtovic A , et al. Funding pandemic prevention, preparedness, and response through partnership models. Lancet Public Health. 2023;8(1):e13. doi:10.1016/S2468-2667(22)00291-2 36493790

[9] Africa CDC . Statement from Africa CDC on the Pandemic Fund . Africa CDC Accessed on September 27, 2023. https://africacdc.org/news‐item/statement‐from‐africa‐cdc‐on‐the‐pandemic‐fund1/

[10] Germán Velásquez . Where Does Global Health Funding Come from and Where Does It Go? . Germán Velásquez. Accessed on September 27, 2023. https://www.southcentre.int/wp‐content/uploads/2023/04/RP176_WHERE‐DOES‐GLOBAL‐HEALTH‐FUNDING‐COME‐FROM‐AND‐WHERE‐DOES‐IT‐GO_EN.pdf

